# Imbalanced Learning Based on Logistic Discrimination

**DOI:** 10.1155/2016/5423204

**Published:** 2016-01-04

**Authors:** Huaping Guo, Weimei Zhi, Hongbing Liu, Mingliang Xu

**Affiliations:** ^1^School of Computer and Information Technology, Xinyang Normal University, Xinyang 464000, China; ^2^School of Information Engineering, Zhengzhou University, Zhengzhou 450000, China

## Abstract

In recent years, imbalanced learning problem has attracted more and more attentions from both academia and industry, and the problem is concerned with the performance of learning algorithms in the presence of data with severe class distribution skews. In this paper, we apply the well-known statistical model logistic discrimination to this problem and propose a novel method to improve its performance. To fully consider the class imbalance, we design a new cost function which takes into account the accuracies of both positive class and negative class as well as the precision of positive class. Unlike traditional logistic discrimination, the proposed method learns its parameters by maximizing the proposed cost function. Experimental results show that, compared with other state-of-the-art methods, the proposed one shows significantly better performance on measures of recall, *g*-mean, *f*-measure, AUC, and accuracy.

## 1. Introduction

Recently, class imbalance problem, also called skewed or rare class problem, has drawn a significant number of interests in academia, industry, and government. For the two-class case, this problem is characterized as having many more examples of one class (majority class or negative class) than the other (minority class or positive class) [1–3]. In many real-world applications, the correct prediction of examples in positive class is often more meaningful than the contrary case. For example, in cancer detection, most patients belong to common disease, rare patients may have cancer, and how to effectively recognize cancer patients is very meaningful. However, conventional classification methods such as C4.5, naive bayes, and neural network, try to pursue a high accuracy by assuming that all classes have similar size, which leads to the fact that the rare class examples are often overlooked and misclassified to majority class [4, 5].

Many approaches have been proposed to tackle this problem, which can be roughly categorized into three levels: data preprocessing level, algorithm learning level, and prediction postprocessing level. For the data preprocessing level, the algorithms focus more on examples with positive class through one of the three approaches: (1) the algorithms running on the rebalanced data sets obtained by manipulating the data space [6, 7] such as undersampling technique and oversampling one, (2) actively selecting the more valuable examples to learn models and leaving the ones with less information to improve models' performance [8, 9], and (3) weighting data space using information concerning misclassification costs to avoid costly errors [10]. The approaches at the algorithm learning level try to adjust existing classifier learning algorithms such that the learned models are biased towards correctly classifying positive class examples, such as two-phase rule induction [11] and one-class learning. Existing approaches at prediction postprocessing level try to focus more on positive class by moving a decision threshold [12] or minimizing a cost function [13].

In this paper, we reconsider the imbalanced problem at algorithm level and propose a novel method called ILLD (Imbalanced Learning Based on Logistic Discrimination) to tackle the problem. The motivation is inspired by the following observation: there are very few researches studying the logistic discrimination on the class imbalanced problem, although it has many merits including understandability, solid theoretical basics, and, most importantly, high generalization ability. Unlike the traditional logistic discrimination, ILLD achieves high performance on imbalanced data by maximizing the proposed cost function APM (Accuracy-Precision Based Metric) which takes into account the accuracies of both positive class and negative class as well as the precision of positive class. Experimental results show that ILLD can much better boost the performance of logistic discrimination on measures of recall, *f*-measure, *g*-mean, and AUC while keeping its high performance on accuracy. Compared with other state-of-the-art classification methods, ILLD shows a much better performance.

The rest of this paper is organized as follows: after presenting related work in Section 2, Section 3 describes the proposed imbalanced learning method; Section 4 presents the experimental results; and, finally, Section 5 concludes this work.

## 2. Related Work

### 2.1. Imbalanced Learning

Technically speaking, the data set which exhibits an unequal distribution between its classes can be considered imbalanced or skewed. However, in the community, only the data sets corresponding to the ones exhibiting extreme imbalances are treated as imbalanced data sets. There are two forms of imbalance, namely, within-class imbalance and between-class imbalance. For the within-class imbalance, some subconcepts exist in limited examples, which increase the difficulty of correctly classifying examples. With respect to the between-class imbalance, one class extremely out-represents another [1, 2]. Usually, the second form of imbalance is often discussed in community.

There are many factors that influence the modeling of a capable classifier when facing rare events. Examples include the skewed data distribution which is considered to be the most influential factor, small sample size, separability, and existence of within-class subconcepts [14].

The skewed data distribution is often denoted by imbalance degree which is the ratio of the sample size of the positive class to that of the negative class. Reported studies indicate that a relatively balanced distribution usually attains a better result. However, to what imbalance degree the class distribution deteriorates the classification performance cannot be stated explicitly, since other factors such as sample size and separability also affect performance [1, 2, 14].

Small sample size means the sample size is limited; uncovering regularities inherent in small class is unreliable. In [15], the authors suggest that the imbalanced class distribution may not be a hindrance to classification by providing a large enough data set.

The difficulty in separating the rare class from the prevalent class is the key issue of the imbalanced problem. Assuming that there exist highly discriminative patterns among each class, then not very sophisticated rules are required to distinguish class objects. However, if patterns among each class are overlapping, discriminative rules are hard to be induced [1, 2, 14].

Within-class concepts mean that a single class is composed of various subclusters or subconcepts. Instances of a class are collected from different subconcepts. These subconcepts do not always contain the same number of instances. The presence of within-class concepts worsens the imbalance distribution problem [14]. In general, we only consider imbalanced data distribution in imbalanced learning and fix other factors.

### 2.2. Logistic Discrimination

Logistic discrimination, also called logistic regression, is a typical probability statistical classification model [16], which has been widely used in many fields such as medical domain and social surveys because of its understandability, solid theoretical basics, and, most importantly, high generalization ability. For the two-class case, logistic discrimination is defined as

(1) py=+ ∣ xi=δwTxi,

(2) py=− ∣ xi=1−py=+ ∣ xi=1−δwTxi,

where *δ*(*a*) is the logistic sigmoid function defined as

(3) $$\begin{array}{c} \delta \left(\;a\;\right)=\frac{1}{1+\exp\left(a\right)}. \end{array}$$

For a given data set *D* = {(**x**_*i*_, *y*_*i*_)∣*i* = 1,2,…, *N*}, where *y*_*i*_ ∈ {+, −} is the label associated with example **x**_*i*_, the likelihood function of this model can be written as

(4) py ∣ w=∏i=1Npyi=+ ∣ xiσi1−pyi=+ ∣ xi1−σi,

where *σ*_*i*_ = 0 if *y*_*i*_ = + and 1 otherwise. Defining a cost function by taking the negative logarithm of the likelihood, we have the cross-entropy error function in the form

(5) Lw=−ln⁡py ∣ w=∑i=1Nσiln⁡pyi=+ ∣ xi+1−σiln⁡1−pyi=+ ∣ xi.

The logistic discrimination uses (5) as the cost function; however, it is not suitable for the class imbalanced problem because the cross-entropy error function defined in (5) does not consider the importance of each class. To handle this problem, a novel cost function called APM (Accuracy-Precision Based Metric) is proposed, which takes into account the accuracies of both positive and negative classes as well as the precision of positive class. For more details refer to Section 3.

### 2.3. Strategies to Handle Imbalanced Problem

The imbalanced problems rise from the scarce representation of the most important examples, which leads to the fact that the learned models tend to focus more on normal examples, overlooking the rare class examples. Many approaches have been proposed to handle the problem, which can be mainly grouped into the following three categories.

- *Data preprocessing based strategy*. These techniques preprocess the given imbalanced data set to change the data distribution such that standard learning algorithms focus more on the cases that are relevant for the user. Reported studies of preprocessing data sets can be categorized into three types: resampling, active learning, and weighting the data space. The object of resampling techniques is to rebalance the class distribution by resampling the data space. Commonly used resampling methods include randomly of informatively undersampling instances in negative class [6], randomly oversampling examples of positive class, oversampling based on cluster algorithm [17, 18], and oversampling the positive class by creating new synthetic instances [7]. Resampling data space technique is often used to deal with imbalanced learning problems, but the real class distribution is always unknown and differs from data to data. Active learning is to actively select the more valuable examples to learn models and leave the ones with less information to improve models' performance by interacting with the user. Several approaches based on active learning have been proposed. For example, Ertekin [9] presented an adaptive oversampling algorithm called VIRTUAL (Virtual Instances Resampling Technique Using Active Learning) to generate synthetic examples for the positive class during the training process, Mi [19] developed a method that combines SMOTE and active learning with SVM, and so on. The strategies of weighting the data space aim to modify the training data set distribution using information concerning the misclassification costs, such that Wang and Japkowicz [10] combined an ensemble of SVM with asymmetric misclassification costs.
- *Algorithm based strategy*. It modifies existing classifier learning algorithms such that the learned models are biased towards the cases that are more concerned by the user. Many algorithms based imbalanced learning approaches have been proposed; for example, Cao et al. [20] presented a framework for improving the performance of cost-sensitive neural networks that adopts Particle Swarm Optimization for optimizing misclassification cost, feature subset, and intrinsic structure parameters; Alejo et al. [21] proposed two strategies for dealing with imbalanced domains using RBF neural networks which include a cost function in the training phase.
- *Prediction postprocessing based strategy*. The approaches of the strategy learn a standard model on the original data set and only modify the predictions of the learned model according to the user references and the imbalance of the data set. There exist two main types of solutions: threshold method and cost-sensitive postprocessing. For the former, each example is associated with a score which expresses the degree to which an example is a member of a class. Based on the scores, a threshold is used to generate different classifiers by varying the threshold for an example belonging to a class [12]. With respect to the latter, several methods exist for making models cost-sensitive in a post hoc manner. This type of strategy was mainly explored for classification tasks and aims at changing only the model predictions for making it cost-sensitive [13].

In this paper, we propose a novel algorithm based imbalanced learning method to improve the performance of the logistic discrimination. Besides, we apply sampling techniques to the logistic discrimination to enhance its performance. Two widely used sampling techniques are selected: random undersampling and oversampling. The corresponding experimental results are presented in Section 4.

## 3. Imbalanced Learning Based on Logistic Discrimination

### 3.1. Accuracy-Precision Based Metric

The traditional logistic discrimination learns its parameters by maximizing the cross-entropy error function defined in (5). However, this approach ignores the diverse costs of classes, which leads to the fact that the learned models have low performance on the positive classes. To tackle this problem, a novel cost function is proposed to guarantee that the learned models perform well on both positive class and negative class. The relevant symbols are defined as follows.

Define *p*_*j*_ and ${\overset{-}{p}}_{j}$ as follows:

(6) pj=∑xi∈Cjpij,p−j=Nj−pj,

where *p*_*ij*_ = *p*(**y**_*i*_ = *j*∣**x**_*i*_) is defined by (1) or by (2). From (6), we have that *p*_*j*_ is the estimation of the number of examples correctly classified as class *j* (corresponding to *n*_*j*_) and ${\overset{-}{p}}_{j}$ is the estimation of number of examples with class *j* incorrectly classified. For two-class problem, we have

(7) p−+=∑xi∈C+pi−,p−−=∑xi∈C−pi+.

Let class “+” be the positive class as used before; then the cost function APM is defined as

(8) $$\begin{array}{c} \text{APM}=\frac{p_{+}}{N_{+}}+\frac{p_{-}}{N_{-}}+\frac{p_{+}}{p_{+}+{\overset{-}{p}}_{-}}. \end{array}$$

Since *p*_*j*_ is the number estimation of examples being correctly classified as class *j* and ${\overset{-}{p}}_{j}$ is that of the ones being incorrectly classified as aforementioned, APM is the estimation of the following equation:

(9) Ln+N++n−N−+n+n++N−−n−=accuracy++recall−+precision+,

where accuracy_+_ is the accuracy (or recall) of positive class (+). Similarly, accuracy_−_ is the accuracy (or recall) of negative class (−) and precision_+_ is the precision of positive class (+). More details about these measures are discussed in Section 4.2. In this way, RPM considers all the three factors: the precision of minority class and the recall of both minority class and majority class.

Taking the gradient of APM (see (8)) with respect to **w** results in

(10) ∇APM=1N+∂p+∂w+1N−∂p−∂w+1p++p−−2p−−∂p+∂w+p+∂p−−∂w,

where

(11) ∂∂wp+∑xi∈C+∂∂wpyi=+ ∣ xi=∑xi∈C+pi+pi−xi,

(12) ∂∂wp−−∑xi∈C−∂∂wpyi=+ ∣ xi=∑xi∈C+pi+pi−xi;

similarly,

(13) $$\begin{array}{c} \frac{\partial }{\partial w}p_{-}=-\sum_{x_{i}\in C_{-}}p_{i+}p_{i-}x_{i}. \end{array}$$

Combining (11), (12), (13), and (10), we have that the gradient of APM defined by (8) is

(14) ∇APM=∑xi∈C+pi+pi−xiN+−∑xi∈C−pi+pi−xiN−+∑xi∈C+pi+pi−xip++p−−−p+∑xi∈C−pi+pi−xip++p−−2.

The proposed method for the imbalanced problem uses a quasi-Newton method BFGS which uses (14) as base function for learning its parameters. For more details refer to Section 3.2.

### 3.2. Algorithm

Based on the cost function APM proposed in Section 3.1, a novel imbalanced learning approach called ILLD (Imbalanced Learning Based on Logistic Discrimination) is proposed to tackle data imbalance. ILLD uses quasi-Newton method BFGS [22–24] to maximize the cost function to learn parameters, where BFGS is an iterative process. Formally, the iterative process is as follows:

(15) $$\begin{array}{c} w^{\left(k+1\right)}=w^{\left(k\right)}-\lambda H^{\left(k\right)}\nabla {\text{APM}}^{\left(k\right)}, \end{array}$$

where *λ* is the step length along with the Newton direction of the *k*th iteration and **H**_*k*_ is the approximate Hessian matrix calculated by

(16) Hk+1=Hk+1+qkTHkqkpkTqkpkpkTpkTqk−pkqkTHk+HkqkpkTpkTqk,

where

(17) pk=wk+1−wk,qk=∇APMk+1−∇APMk.

The details about the learning process of ILLD are shown in Algorithm 1. ILLD firstly initializes **w** ^(0)^ randomly and **H** ^(0)^ to be unit matrix of which the value of each diagonal element is equal to 1 and 0 for others (lines 1~2) and calculates **w** ^(1)^ using (11) based on **w** ^(0)^ and **H** ^(0)^ (lines 3~4). Then ILLD optimizes the cost function ARM to find out the best parameter vector **w** (lines 4~11). Specifically, for the *k*th iteration, ILLD calculates the gradients of ARM^(*k*)^ as **g**_*k*_ using (14) and, based on **g**_*k*_ and **g**_*k*−1_, updates **p** ^(*k*−1)^ and **q** ^(*k*−1)^ using (17) (lines 8~9). Then, it updates **H** ^(*k*)^ using (16) (line 10) and, finally, updates **w** ^(*k*+1)^ using (15) (line 11). The convergence rate of ILLD is *O*(*n* ^2^) [22–24] and the stopping condition is that the absolute of the difference between the values calculated by (15) for two consecutive iterations is not larger than 0.001 (line 13).

### 3.3. Discussion

Unlike traditional logistic discriminations which only consider the overall performances, ILLD takes into account more factors through the accuracy-precision based metric. Indeed, this criterion involves the accuracies (or recalls) of both positive class and negative class as well as the precision of positive class, which result from the prediction confusion matrix (discussed in Section 4.2). Thus ILLD considers not only the overall performance of logistic discrimination but also the performance on each class.

Considering only the former terms of ARM defined by (8), we have

(18) $$\begin{array}{c} \frac{\text{APM}}{2}=\frac{\left(p_{+}/N_{+}+p_{-}/N_{-}\right)}{2}. \end{array}$$

Similarly, considering only the former terms of (9), we have the evaluation measure of AUC [2] as shown in the following:

(19) $$\begin{array}{c} \frac{L}{2}=\frac{0.5n_{+}}{N_{+}}+\frac{0.5n_{-}}{N_{-}}=\frac{{\text{accuracy}}_{+}+{\text{accuracy}}_{-}}{2}. \end{array}$$

Therefore, the proposed measure (without considering the last term) is the estimation of AUC. Besides, comparing (18), (19), and the evaluation of *g*-mean defined as

(20) $$\begin{array}{c} g\text{-mean}=\sqrt{{\text{accuracy}}_{+}\times {\text{accuracy}}_{-}} \end{array}$$

we conclude that the proposed measure (without considering the last term) uses the arithmetic mean of accuracies (or recalls) of both positive class and negative class instead of the geometric mean as the cost function to supervise the learning process of the logistic discriminations.

Omitting the second term of both (11) and (12), then

(21) APMp+N++p+p++p−−,Ln+N++n+n++N−−n−=accuracy++precision+.

We observe from (21) that the proposed cost function is the metric that combines the accuracy (or recall) and precision of positive class together as *f*-measure does.

## 4. Experiments

### 4.1. Data Sets and Experimental Setup

The 14 data sets utilized in this paper are randomly selected from the UCI repository [25]. Of these data sets, breast-Wisconsin, hepatitis, horse-colic, and ionosphere are imbalanced 2-class data sets. Others are 2-class imbalanced data sets derived from multiclass data sets by treating one class of a multiclass data set as the positive class while treating the union of all other classes as the negative class [26]. The imbalanced degree of these data sets varies from 0.0376 (highly imbalanced) to 0.3696 (only slightly imbalanced), where imbalanced degree is defined as the ratio of the sample size of the positive class to that of the negative class. The details about the data sets are shown in Table 1, where #Degree is the imbalance degree, #Exs is the size of data sets, #Attrs is the number of attributes, and #Cls is the number of classes. For each data set, a 5 × 2-fold cross-validation [27] is performed.

To evaluate the performance of ILLD, we compare it with LD, LD-US, and LD-OS, where LD denotes that traditional logistic discrimination (cross-entropy error function is treated as cost function) is simply applied to imbalanced problem and LD_US and LD_OS denote that LD runs on data sets obtained by undersampling and by oversampling the training data sets, respectively. Here, the prediction postprocessing approaches such as threshold method [12] are not used for comparisons, since the study in [28] concluded that the operations of moving the decision threshold, applying a sampling strategy, and adjusting the cost matrix produce classifiers with the same performance.

### 4.2. Evaluation Metrics

Evaluation metric is extremely essential to assessing the effectiveness of an algorithm and, traditionally, accuracy is the most frequently used one. Considering two-class classification problem and letting “+” and “−” be the positive and negative classes, respectively, as aforementioned, then examples can be categorized into four groups after a classification process as denoted in the confusion matrix presented in Table 2, and thus the accuracy is defined as

(22) $$\begin{array}{c} \text{accuracy}=\frac{\text{TP}+\text{TN}}{\text{TP}+\text{TN}+\text{FP}+\text{FN}}. \end{array}$$

However, the evaluation metrics used for the balanced problem is very different from that used for the imbalanced one, and accuracy is inadequate for imbalanced learning. In lieu of accuracy, other assessment metrics including recall, precision, *f*-measure, and *g*-mean are frequently adopted in the research community to evaluate the performance of models on imbalanced learning problems. These metrics are designed based on the accuracy of both positive class and negative class and the precision of negative class, specifically:

(23) accuracy+=recall=TPTP+FN,accuracy−=TNTN+FP,precision+=precision=TPTP+FP.

Then, *f*-measure and *g*-mean are defined as

(24) f-measure1+ρ2×recall×precisionρ2×recall+precision=1+ρ2×accuracy+×precision+ρ2×accuracy++precision+,g-meanaccuracy+×accuracy−,

where *ρ* is a coefficient to adjust the relative importance of precision versus recall (usually, *ρ* = 1).

From (24), *f*-measure combines recall and precision as a measure of the effectiveness of classification in terms of a ratio of the weighted importance on either recall (accuracy_+_) or precision (precision_+_) as determined by the user. So, *f*-measure represents a harmonic mean between recall and precision. Like *f*-measure, *g*-mean is also a metric which evaluates models' performance by considering two metrics; specifically, *g*-mean measures the balanced performance of a classifier using the geometric mean of the recall of positive class and that of negative class.

In the case of the soft-type classifiers, that is, classifiers that output a continuous numeric value to represent the confidence of an example belonging to the predicted class, AUC is a commonly used measure to evaluate models' performances, which can be calculated by

(25) $$\begin{array}{c} \text{AUC}=\frac{{\text{accuracy}}_{+}+{\text{accuracy}}_{-}}{2}. \end{array}$$

The AUC allows the evaluation of the best model on average.

In this paper, we employ accuracy, recall, *f*-measure, *g*-mean, and AUC to evaluate the classification performance on imbalanced data sets. Though accuracy is inadequate to evaluate the classification performance, poor accuracy means a bad classifier. An efficient classifier should improve recall, *f*-measure, *g*-mean, or AUC without decreasing accuracy.

### 4.3. Experimental Results

**Figure 1:**
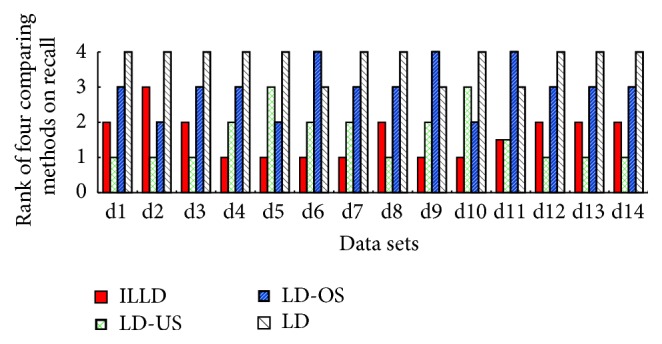
The ranks of ILLD, LD-US, LD-OS, and LD on measure of recall.

**Figure 2:**
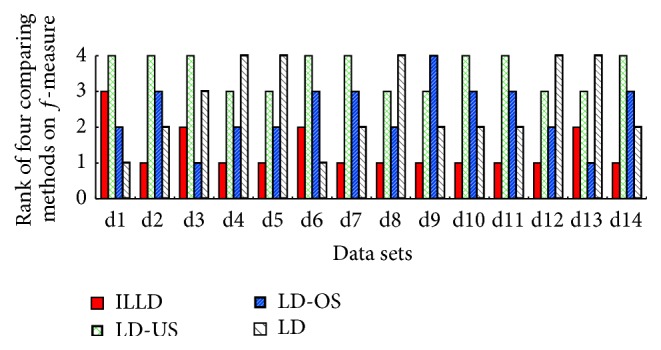
The ranks of ILLD, LD-US, LD-OS, and LD on *f*-measure.

**Figure 3:**
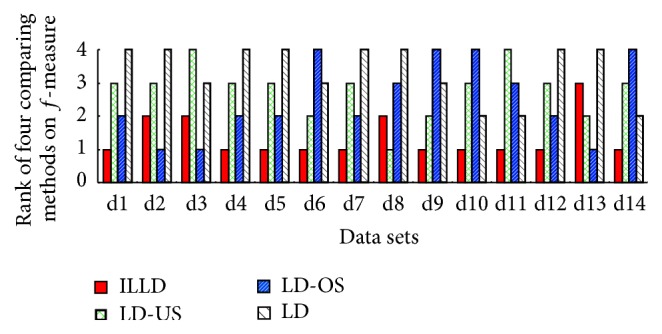
The ranks of ILLD, LD-US, LD-OS, and LD on *g*-mean.

**Figure 4:**
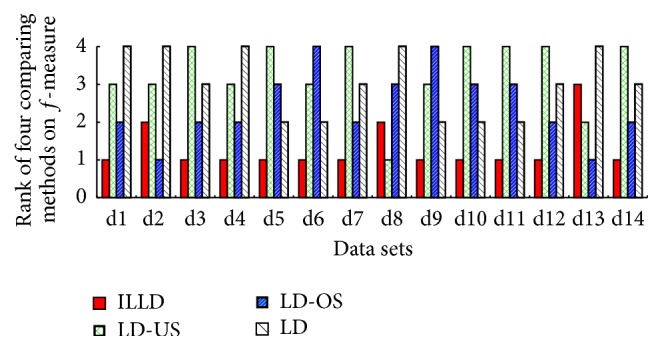
The ranks of ILLD, LD-US, LD-OS, and LD on AUC.

To evaluate the performance of ILLD (the proposed method), ILLD is compared with LD, LD-US, and LD-OS (for more details about LD, LD-US, and LD-OS refer to Section 4.1). The corresponding results are reported in both tables and figures, where Tables 3, 4, 5, 6, and 7 report the results of the four comparing methods on the measures of accuracy, recall, *g*-mean, *f*-measure, and AUC and Figures 1, 2, 3, and 4 report the ranks of the methods on recall, *g*-mean, *f*-measure, and AUC. In these tables, a bullet (an open circle) next to a result indicates that ILLD significantly outperforms (is outperformed by) the respective method (column) for respective data set (row) in pairwise *t*-test at 95% significance level. The last rows in these tables are the average results. The ranks of these methods shown in Figures 1, 2, 3, and 4 are calculated as follows [29, 30]: on a data set, the best performing algorithm gets the rank of 1.0, the second best performing algorithm gets the rank of 2.0, and so on. In case of ties, average ranks are assigned.

Table 3 reports the accuracies of ILLD, LD-US, LD-OS, and LD. As shown in Table 3, ILLD outperforms LD on three data sets and is outperformed by LD on four ones for *t*-test at 95% significant level. Moreover, the average accuracy of ILLD is 1.19 percentage points lower than the one of LD. The results are acceptable although LD is better than ILLD since we focus on imbalanced learning of which accuracy is not an ideal metric to evaluate its performance. Compared to LD-US and LD-OS, ILLD shows significantly better performances on 13 and 7 out of the 14 data sets, respectively, and 11.47 and 2.74 percentage points higher performance on the average accuracies, respectively.

Table 4 and Figure 1 show the summarizing results and the ranks of the four comparing methods on measure of recall, respectively. From Table 4, ILLD significantly outperforms LD on 10 out of the 14 data sets, and the average recall of ILLD is 0.1454 higher than LD (recall ∈[0,1]). These results indicate that the proposed cost function APM is appropriate for imbalanced problem and thus ILLD can improve the performance of logistic discrimination on positive class while keeping its high performance on the measure of accuracy. Also, ILLD performs comparable to LD-US and outperforms LD-OS. Specifically, ILLD significantly outperforms LD-US and LD-OS on 2 and 3 data sets, respectively, and is only outperformed by LD-US on one data set. Besides, from Figure 1, we can see that the average ranks of ILLD, LD-US, LD-OS, and LD are 1.61, 1.61, 3.0, and 3.78, respectively. Combining with the results in Table 3, we have that LD-US achieves a high recall by sacrificing the high performance of logistic discrimination on accuracy.

Table 5 and Figure 2 illustrate the summarizing results and the ranks of ILLD, LD-US, LD-OS, and LD on *f*-measure, respectively. From Table 5, ILLD shows much better performance comparing to LD-US, LD-OS, and LD. Specifically, ILLD significantly outperforms them on 11, 7, and 6 out the 14 data sets, respectively, and is only outperformed by LD-OS on one data set. Moreover, Figure 2 shows that ILLD wins on 14, 12, and 11 out of the 14 data sets comparing to LD-US, LD-OS, and LD, respectively. Besides, the *f*-measure of ILLD ranks first on 10 data sets.

*g*-mean summaries and the corresponding ranks of ILLD, LD-US, LD-OS, and LD are reported in Table 6 and Figure 3. Similar to the results shown in Table 4 and Figure 2, Table 5 shows that ILLD significantly outperforms LD-US, LD-OS, and LD on 7, 6, and 10 out of 14 data sets, respectively, and Figure 3 shows that ILLD wins on 13, 11, and 14 data sets comparing to the four methods, respectively. Besides, ILLD ranks first with average rank of 1.36, followed by LD-OS (2.78), LD-US (2.5), and LD (3.36).

Table 7 and Figure 4 depict AUCs and the ranks of the four comparing methods, respectively. On the 14 data sets, ILLD wins (significantly wins) on 11 (6), 12 (7), and 11 (6) out of 14 data sets comparing to LD-OS, LD-US, and LD. The average AUCs (ranks) of ILLD, LD-OS, LD-US, and LD are 0.8114 (1.29), 0.7559 (3.29), 0.7649 (2.43), and 0.7562 (3), respectively.

## 5. Conclusion

In this paper, we first construct a novel cost function called APM (Accuracy-Precision Based Metric) which considers the accuracies of both positive class and negative class as well as the precision of positive class and then propose a method called ILLD (Imbalanced Learning Based on Logistic Discrimination) to handle data imbalances. Also, we apply undersampling and oversampling to improve the performance of logistic discrimination on the imbalanced problem. Experimental results show that these methods can significantly improve the performance of logistic discrimination on positive class, and ILLD presents significantly better performances compared to other advanced methods on measures of recall, *f*-measure, and *g*-mean, while keeping the high performance of logistic discrimination on accuracy.

## Figures and Tables

**Algorithm 1:**
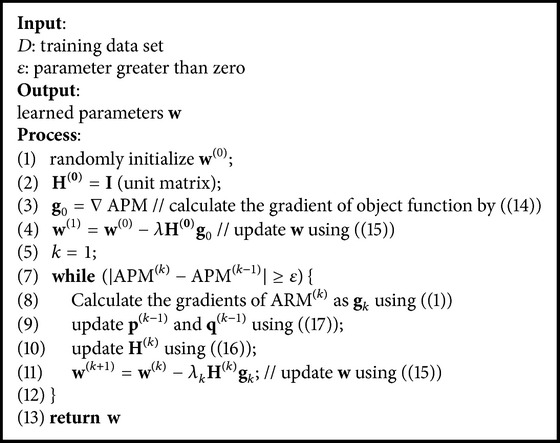
ILLD.

**Table 1:** The description of experimental data sets.

	Data sets	#Degree	#Exs	#Attrs	#Cls
d1	Anneal	0.0445	898	39	6
d2	Audiology	0.0885	226	70	24
d3	Autos	0.1073	205	26	7
d4	Breast-Wisconsin	0.3448	699	11	2
d5	Breast-cancer	0.2972	286	10	2
d6	Car	0.0376	1728	7	4
d7	Flag	0.0876	194	28	6
d8	Glass	0.0607	214	10	7
d9	Hepatitis	0.2065	155	20	2
d10	Horse-colic	0.3696	368	23	2
d11	Ionosphere	0.3589	351	35	2
d12	Solar-flare	0.0781	333	11	8
d13	Splice	0.2404	3190	62	3
d14	Yeast	0.0134	1480	9	10

**Table 2:** Confusion matrix.

	Predicted as positive (+)	Predicted as negative (−)
Actually positive (+)	TP	FN
Actually negative (−)	FP	TN

**Table 3:** The accuracies and standard errors of ILLD, LD-US, LD-OS, and LD.

Data set	ILLD	LD-US	LD-OS	LD
d1	94.39 ± 4.41	85.84 ± 6.06^•^	95.35 ± 3.55	97.46 ± 1.15
d2	91.86 ± 2.94	82.57 ± 7.19^•^	88.94 ± 4.01	91.86 ± 3.12
d3	85.36 ± 5.69	73.69 ± 7.63^•^	86.54 ± 6.08	86.25 ± 5.51
d4	96.71 ± 0.36	94.25 ± 2.69^•^	95.08 ± 0.84^•^	93.62 ± 1.42^•^
d5	66.78 ± 2.80	58.25 ± 3.94^•^	57.20 ± 4.09^•^	67.69 ± 2.63
d6	98.19 ± 0.46	88.75 ± 3.07^•^	97.43 ± 0.88^•^	98.61 ± 0.39°
d7	80.21 ± 4.03	61.75 ± 8.23^•^	82.47 ± 4.79	83.61 ± 4.30
d8	91.59 ± 6.28	83.46 ± 10.21^•^	91.40 ± 4.98	93.64 ± 1.31
d9	78.97 ± 4.13	69.80 ± 5.93^•^	77.30 ± 5.42	78.46 ± 5.16
d10	80.43 ± 2.66	70.71 ± 3.89^•^	74.62 ± 2.35^•^	77.66 ± 3.34^•^
d11	86.78 ± 1.99	82.05 ± 3.74^•^	83.19 ± 2.95^•^	84.21 ± 3.92
d12	72.43 ± 5.84	51.06 ± 9.56^•^	66.91 ± 6.56^•^	90.87 ± 1.31°
d13	93.99 ± 1.19	93.71 ± 0.77	96.37 ± 0.28°	90.43 ± 1.96^•^
d14	99.08 ± 0.20	60.27 ± 6.91^•^	85.63 ± 5.97^•^	99.04 ± 0.18

Average	86.91	75.44	84.17	88.10

**Table 4:** The recalls and standard errors of ILLtD, LD-US, LD-OS, and LD.

Data set	ILLD	LD	LD-US	LD-OS
d1	0.8950 ± 0.1141	0.9350 ± 0.1001	0.8550 ± 0.0956	0.7600 ± 0.0937^•^
d2	0.7800 ± 0.1619	0.8600 ± 0.1897	0.8400 ± 0.1430	0.7200 ± 0.2201
d3	0.7364 ± 0.2326	0.7727 ± 0.2278	0.7091 ± 0.1342	0.6273 ± 0.1890
d4	0.9709 ± 0.0143	0.9343 ± 0.0722	0.9228 ± 0.0286^•^	0.8755 ± 0.0382^•^
d5	0.6096 ± 0.0806	0.5413 ± 0.0550^•^	0.6003 ± 0.0645	0.3693 ± 0.0670^•^
d6	0.9755 ± 0.0535	0.9323 ± 0.1121	0.6619 ± 0.0996^•^	0.8311 ± 0.1227^•^
d7	0.4833 ± 0.1838	0.4764 ± 0.2075	0.3556 ± 0.1336	0.3167 ± 0.2010^•^
d8	0.7786 ± 0.1831	0.9048 ± 0.1139	0.6310 ± 0.2457	0.4429 ± 0.2580^•^
d9	0.7125 ± 0.1291	0.6750 ± 0.1133	0.5375 ± 0.1070^•^	0.5687 ± 0.1486
d10	0.7574 ± 0.0593	0.7015 ± 0.0416^•^	0.7412 ± 0.0506	0.7044 ± 0.0512^•^
d11	0.6778 ± 0.0599	0.6778 ± 0.0608	0.6683 ± 0.0696	0.6698 ± 0.0664
d12	0.3923 ± 0.0674	0.5077 ± 0.1926	0.3692 ± 0.1013	0.0846 ± 0.0437^•^
d13	0.9570 ± 0.0232	0.9744 ± 0.0087	0.9429 ± 0.0088	0.8404 ± 0.0575^•^
d14	0.5500 ± 0.1080	0.7300 ± 0.2163°	0.5000 ± 0.2160	0.4300 ± 0.0949^•^

Average	0.7340	0.7588	0.6668	0.5886

**Table 5:** The *f*-measures and standard errors of ILLD, LD, LD-US, and LD-OS.

Data set	ILLD	LD-US	LD-OS	LD
d1	0.6446 ± 0.1734	0.3955 ± 0.1161^•^	0.6587 ± 0.1788	0.7322 ± 0.1060
d2	0.6335 ± 0.0855	0.4778 ± 0.0883^•^	0.5826 ± 0.1003	0.6062 ± 0.1561
d3	0.5207 ± 0.1391	0.3884 ± 0.0974^•^	0.5475 ± 0.1507	0.5019 ± 0.1680
d4	0.9532 ± 0.0046	0.9170 ± 0.0416^•^	0.9281 ± 0.0130^•^	0.9042 ± 0.0221^•^
d5	0.5204 ± 0.0353	0.4353 ± 0.0302^•^	0.4546 ± 0.0399^•^	0.4025 ± 0.0478^•^
d6	0.8047 ± 0.0367	0.3926 ± 0.0757^•^	0.6637 ± 0.0888^•^	0.8149 ± 0.0655
d7	0.2975 ± 0.0961	0.1764 ± 0.0681^•^	0.2704 ± 0.1140	0.2391 ± 0.1156
d8	0.5621 ± 0.1615	0.4358 ± 0.1162	0.4809 ± 0.1501	0.4170 ± 0.1798
d9	0.5825 ± 0.0667	0.4812 ± 0.0724^•^	0.4966 ± 0.0936^•^	0.5193 ± 0.1135
d10	0.7407 ± 0.0348	0.6396 ± 0.0387^•^	0.6833 ± 0.0245^•^	0.6999 ± 0.0412^•^
d11	0.7853 ± 0.0378	0.7314 ± 0.0433^•^	0.7392 ± 0.0528	0.7526 ± 0.0604
d12	0.1837 ± 0.0186	0.1358 ± 0.0363^•^	0.1487 ± 0.0282^•^	0.1242 ± 0.0616^•^
d13	0.8849 ± 0.0188	0.8817 ± 0.0130	0.9259 ± 0.0050°	0.8081 ± 0.0402^•^
d14	0.6157 ± 0.0880	0.0475 ± 0.0133^•^	0.0988 ± 0.0450^•^	0.5455 ± 0.0825^•^

Average	0.6235	0.4669	0.5485	0.5763

**Table 6:** The *g*-means and standard errors of ILLD, LD, LD-US, and LD-OS.

Data set	ILLD	LD	LD-US	LD-OS
d1	0.9170 ± 0.0434	0.8930 ± 0.0693	0.9040 ± 0.0619	0.8636 ± 0.0553^•^
d2	0.8469 ± 0.0823	0.8314 ± 0.0945	0.8628 ± 0.0701	0.8123 ± 0.1334
d3	0.7865 ± 0.1364	0.7391 ± 0.1094	0.7886 ± 0.0882	0.7407 ± 0.1256
d4	0.9679 ± 0.0031	0.9398 ± 0.0374^•^	0.9437 ± 0.0126^•^	0.9204 ± 0.0195^•^
d5	0.6468 ± 0.0311	0.5679 ± 0.0300^•^	0.5783 ± 0.0396^•^	0.5429 ± 0.0421^•^
d6	0.9784 ± 0.0253	0.9068 ± 0.0577^•^	0.8060 ± 0.0596^•^	0.9055 ± 0.0696^•^
d7	0.6199 ± 0.1395	0.5082 ± 0.1867	0.5468 ± 0.1193	0.5003 ± 0.1611^•^
d8	0.8442 ± 0.1140	0.8611 ± 0.0642	0.7530 ± 0.1540	0.6021 ± 0.2597^•^
d9	0.7549 ± 0.0602	0.6858 ± 0.0658^•^	0.6668 ± 0.0748^•^	0.6847 ± 0.0992
d10	0.7926 ± 0.0292	0.7052 ± 0.0362^•^	0.7439 ± 0.0221^•^	0.7587 ± 0.0338^•^
d11	0.8117 ± 0.0328	0.7793 ± 0.0336	0.7845 ± 0.0431	0.7920 ± 0.0465
d12	0.5394 ± 0.0259	0.4880 ± 0.0764^•^	0.4991 ± 0.0575^•^	0.2694 ± 0.1057^•^
d13	0.9455 ± 0.0089	0.9495 ± 0.0064	0.9565 ± 0.0024°	0.8810 ± 0.0321^•^
d14	0.7372 ± 0.0733	0.6512 ± 0.0995^•^	0.6223 ± 0.2299	0.6515 ± 0.0722^•^

Average	0.7992	0.7504	0.7469	0.7089

**Table 7:** The AUCs and standard errors of ILLD, LD, LD-US, and LD-OS.

Data set	ILLD	LD	LD-US	LD-OS
d1	0.9206 ± 0.0397	0.8949 ± 0.0695	0.9065 ± 0.0589	0.8723 ± 0.0485^•^
d2	0.8560 ± 0.0704	0.8412 ± 0.0804	0.8671 ± 0.0664	0.8289 ± 0.1123
d3	0.8021 ± 0.1120	0.7527 ± 0.1009	0.7966 ± 0.0809	0.7590 ± 0.1083
d4	0.9680 ± 0.0031	0.9405 ± 0.0365^•^	0.9441 ± 0.0124^•^	0.9218 ± 0.0186^•^
d5	0.6510 ± 0.0283	0.5706 ± 0.0309^•^	0.5802 ± 0.0397^•^	0.5881 ± 0.0269^•^
d6	0.9788 ± 0.0244	0.9090 ± 0.0560^•^	0.8242 ± 0.0491^•^	0.9116 ± 0.0604^•^
d7	0.6580 ± 0.0922	0.5532 ± 0.0858^•^	0.6128 ± 0.0795	0.6014 ± 0.0844
d8	0.8519 ± 0.1075	0.8672 ± 0.0581	0.7816 ± 0.1258	0.7055 ± 0.1172^•^
d9	0.7611 ± 0.0546	0.6895 ± 0.0634^•^	0.6859 ± 0.0659^•^	0.7047 ± 0.0774
d10	0.7946 ± 0.0282	0.7059 ± 0.0360^•^	0.7452 ± 0.0214^•^	0.7617 ± 0.0329^•^
d11	0.8260 ± 0.0267	0.7891 ± 0.0316^•^	0.7959 ± 0.0368	0.8042 ± 0.0423
d12	0.5724 ± 0.0177	0.5093 ± 0.0616^•^	0.5319 ± 0.0401^•^	0.5315 ± 0.0207^•^
d13	0.9457 ± 0.0087	0.9498 ± 0.0064	0.9566 ± 0.0023°	0.8824 ± 0.0307^•^
d14	0.7734 ± 0.0538	0.6655 ± 0.0990^•^	0.6806 ± 0.1220	0.7140 ± 0.0471^•^

Average	0.8114	0.7599	0.7649	0.7562
